# Assessing Clinical Reasoning: Targeting the Higher Levels of the Pyramid

**DOI:** 10.1007/s11606-019-04953-4

**Published:** 2019-04-25

**Authors:** Harish Thampy, Emma Willert, Subha Ramani

**Affiliations:** 1 Division of Medical Education, School of Medical Sciences, Faculty of Biology, Medicine & Health, University of Manchester, Manchester, UK; 2 Harvard Medical School, Brigham and Women’s Hospital, General Internal Medicine, Department of Medicine, Boston, MA USA

**Keywords:** medical education-assessment method, medical education-assessment/evaluation, medical education-cognition/problem solving, clinical reasoning

## Abstract

Clinical reasoning is a core component of clinical competency that is used in all patient encounters from simple to complex presentations. It involves synthesis of myriad clinical and investigative data, to generate and prioritize an appropriate differential diagnosis and inform safe and targeted management plans.

The literature is rich with proposed methods to teach this critical skill to trainees of all levels. Yet, ensuring that reasoning ability is appropriately assessed across the spectrum of knowledge acquisition to workplace-based clinical performance can be challenging.

In this perspective, we first introduce the concepts of illness scripts and dual-process theory that describe the roles of analytic system 1 and non-analytic system 2 reasoning in clinical decision making. Thereafter, we draw upon existing evidence and expert opinion to review a range of methods that allow for effective assessment of clinical reasoning, contextualized within Miller’s pyramid of learner assessment. Key assessment strategies that allow teachers to evaluate their learners’ clinical reasoning ability are described from the level of knowledge acquisition, through to real-world demonstration in the clinical workplace.

## INTRODUCTION

**Figure 1:**
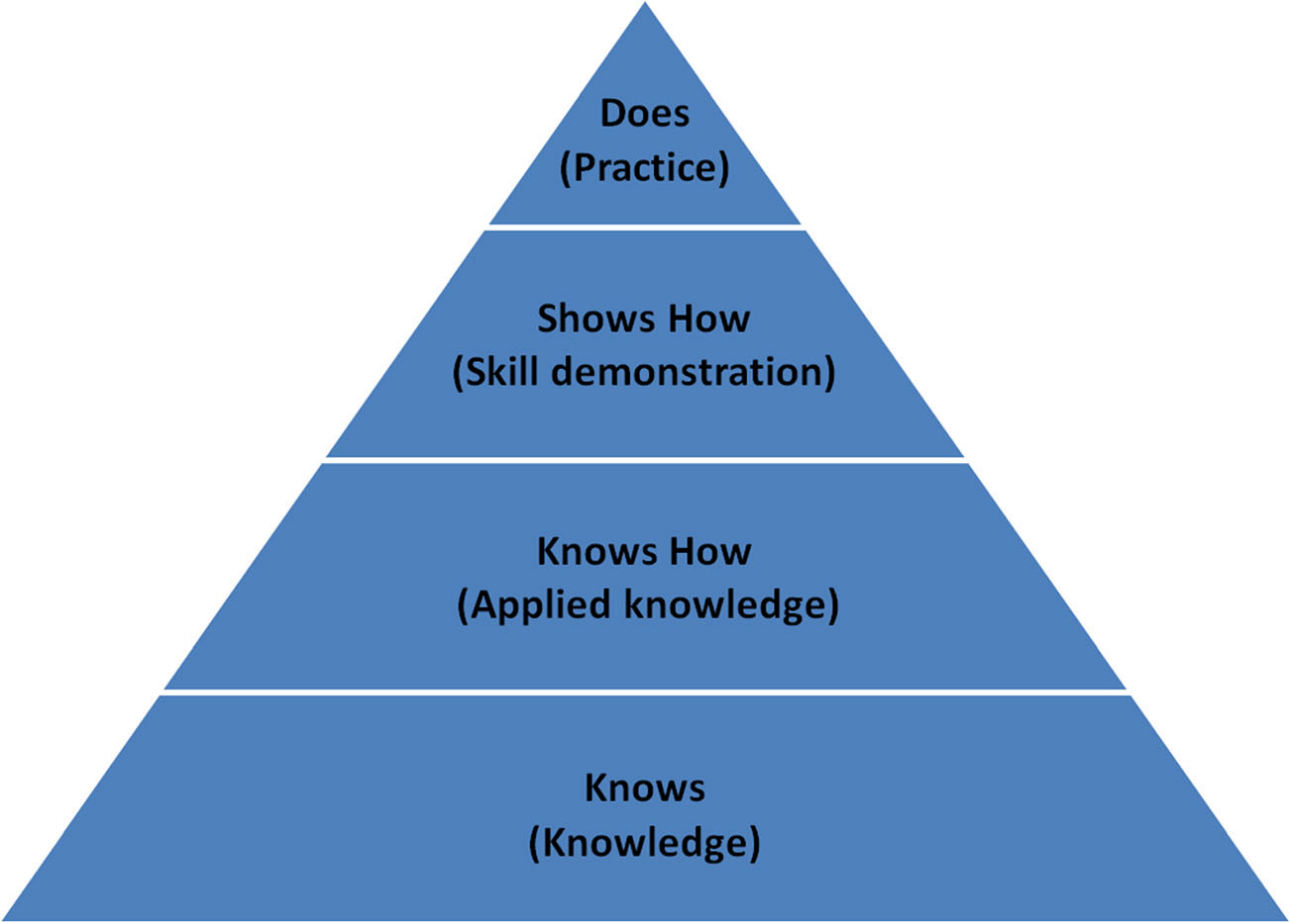
**Miller’s pyramid of clinical competence (supplied also as a .tif file). Adapted from Miller**^2^**.**

Clinical reasoning is the “thinking and decision making processes associated with clinical practice.”^1^ It involves pattern recognition, knowledge application, intuition, and probabilities. It is integral to clinical competency and is gaining increasing attention within medical education. Much has been written about the cognitive processes underpinning reasoning strategies and the myriad ways educators can enhance learners’ clinical reasoning abilities. Literature on*assessing* clinical reasoning, however, is more limited with focus on written assessments targeting the lower levels of “knows” and “knows how” of Miller’s pyramid (Fig. 1).^2^ This article offers a more holistic perspective on assessing clinical reasoning by exploring current thinking and strategies at all levels.

## CLINICAL REASONING MODELS

Although many clinical reasoning models have been proposed, script theory^3^ and dual-process theory^4^ have attracted particular attention among medical educators. Script theory suggests clinicians generate and store mental representations of symptoms and findings of a particular condition (“illness scripts”) with networks created between existing and newly learnt scripts. Linked to this is dual-process theory which suggests that clinical decision making operates within two systems of thinking. System 1 thinking utilizes pattern recognition, intuition, and experience to effortlessly activate illness scripts to quickly arrive at a diagnosis. Conversely, clinicians utilizing system 2 thinking analytically and systematically compare and contrast illness scripts in light of emerging data elicited from history and examination while factoring in demographic characteristics, comorbidity, and epidemiologic data. In this approach, clinicians test probable hypotheses, using additional information to confirm or refute differential diagnoses. Although system 2 thinking requires more cognitive effort, it is less prone to the biases inherent within system 1 thinking.^5^ Several cognitive biases have been characterized^6^ with key examples outlined in Table 1. With increasing experience, clinicians skillfully gather and apply relevant data by continually shifting between non-analytic and analytic thinking.^7^

**Table 1:** **Examples of Cognitive Biases**

Cognitive bias	Description
Anchoring bias	The tendency to over rely on, and base decisions on, the first piece of information elicited/offered
Confirmation bias	The tendency to look for evidence to confirm a diagnostic hypothesis rather than evidence to refute it
Availability bias	The tendency to over rely on, and base decisions on, recently encountered cases/diagnoses
Search satisficing	The tendency to stop searching for other diagnoses after one diagnosis appears to fit
Diagnosis momentum	The tendency to continue relying on an initial diagnostic label assigned to a patient by another clinician
Ambiguity effect	The tendency to make diagnoses for which the probability is known over those for which the probability is unknown

Attempting to assess complex internal cognitive processes that are not directly observable poses obvious challenges. Furthermore, it cannot be assumed that achieving correct final outcomes reflects sound underpinning reasoning. Potential strategies however have been suggested to address these difficulties and are described below at each level of Miller’s pyramid.

## ASSESSING CLINICAL REASONING AT THE “KNOWS” AND “KNOWS HOW” LEVELS

In the 1970s, *patient management problems* (PMPs) were popular and utilized a patient vignette from which candidates selected management decisions.^8^ Originally designed to assess problem-solving strategies, later work suggested PMPs were likely only testing knowledge acquisition.^9^ Often, there was disagreement among experts on the possible correct answer along with poor case specificity (performance on one case poorly predicting performance on another).^10^ Furthermore, experienced clinicians did not always score higher than juniors.^10^ As a result, the use of PMPs has declined.

Subsequently, *script concordance tests* (SCTs) were developed based on the previously mentioned concept of “illness script.”^3^ An example SCT is shown in Text box 1. Examinees are faced with a series of patient scenarios and decide, using a Likert-type scale, whether a particular item (such as a symptom, test, or result) would make a diagnosis more or less likely. Examinees’ answers are compared with those from experts, with weighted scoring applied to responses chosen by more expert clinicians.^11^ SCTs offer reliable assessments (achieving alpha of 0.77–0.82)^11–13^ with agreement from both examiners and candidates that real-world diagnostic thinking is being assessed.^12,14^ They predict performance on other assessments (such as Short Answer Management Problems and Simulated Office Orals),^15^ allow for discrimination across the spectrum of candidate ability,^12^ and show improved performance with increasing experience (construct validity).^12^

**Text Box 1. Example SCT**

A 55-year-old man presents to your clinic with a persistent cough of 6 weeks.

**Table:** 

If you were thinking of:	And then you find:	This diagnosis becomes
Q1: Lung cancer	Patient has smoked 20 cigarettes a day for 30 years	− 2 − 1 0 + 1 + 2
Q2: Drug side effect	Patient started ace inhibitor 6 weeks ago	− 2 − 1 0 + 1 + 2
Q3: COPD	Patient has never smoked	− 2 − 1 0 + 1 + 2

− 2 Ruled out or almost ruled out; − 1 Less likely; 0 Neither more nor less likely; + 1 More likely; + 2 Certain or almost certain

*Key feature questions* (KFQs) require candidates to identify essential elements within a clinical vignette in relation to possible diagnoses, investigations, or management options.^16^ In keeping with SCTs, KFQs similarly demonstrate good face validity, construct validity, and predictive validity of future performance.^16^ In addition, KFQs are thought to have an advantage over SCTs as they can minimize the cueing effect within the latter’s response format.^17^

The *clinical integrative puzzle* (CIP) bases itself on the extended matching question concept but utilizes a grid-like appearance which requires learners to compare and contrast a group of related diagnoses across domains such as history, physical examination, pathology, investigations, and management.^18^ CIPs encourage integration of learning and consolidation of illness scripts and demonstrate good reliability (up to 0.82) but only modest validity.^19^

The *ASCLIRE method* uses computer-delivered patient scenarios that allow learners to seek additional data from a range of diagnostic measures in order to select a final diagnosis from a differential list.^20^ Diagnostic accuracy, decision time, and choice of additional diagnostic data are used to differentiate reasoning abilities. It is estimated that 15 scenarios, over 180 min, would achieve a reliability of 0.7. ASCLIRE is well received by candidates and demonstrates appropriate construct validity, with experts outscoring novices.^20^

More recently, *virtual patients* have been developed with software enabling students to categorize diagnoses as unlikely or not to be missed through illness script–based concept maps.^21^ Although currently proposed for use as a learning tool, future development could offer assessment possibilities.

## ASSESSING CLINICAL REASONING AT THE “SHOWS HOW” LEVEL

Objective structured clinical examinations (OSCEs) are widely accepted as robust assessments of learners’ clinical competencies. From the first papers describing OSCEs in the 1970s^22^ to the multitude of publications since, their ability to assess a range of clinical skills including problem-solving abilities has been emphasized. Despite this stated aim, the literature however is limited to components of clinical competency such as history taking, physical examination, or explanation of diagnoses, with less attention paid to understanding how OSCEs can be used to assess clinical reasoning ability.

Given the paucity of published work in this area, assessment and teaching academics from the lead authors’ institution have worked collaboratively to transform historically used OSCE stations, which often operated on simple pattern recognition, into stations that require analytical system 2 thinking. Table 2 describes strategies that have proven successful.

**Table 2:** **Suggested OSCE design strategies**

Station type	Design strategies
History taking stations	Create simulated patient scripts such that not all possible symptoms are present and/or add in symptoms that may suggest more than one plausible differential diagnosis
	Include end-of-station examiner questions that require candidates to not only name, but also justify, their likely differential diagnoses
	In longer stations, consider stop-start techniques in which candidates are asked at different time points to list their differential diagnoses
Physical examination stations	Design hypothesis-driven or presentation-based examinations (requiring the candidate to conduct an appropriate examination from a stated differential list or short clinical vignette) rather than full system-based examinations^23^
Data interpretation stations	Utilize clinical data (either at once or sequentially) along with a clinical vignette and examiner questions to assess not just the candidate’s data *reporting* ability, but*interpretation* ability in light of the clinical situation described
Explanation stations	Provide clinical results/data requiring candidate interpretation and offering real-world clinical context to base explanations and justifications

Further modifications to the traditional OSCE format replace end-of-station examiner questions with post-encounter forms (PEF, also called progress notes or patient notes) as an inter-station task.^24–28^ Following a typical consultation-based OSCE station (history taking/clinical examination), the candidate is required to write a summary statement, list of differential diagnoses, and, crucial to the assessment of reasoning, their justification for each differential using supporting or refuting evidence obtained from the consultation. Post-encounter forms demonstrate good face validity^24^ and inter-rater reliability through the use of standardized scoring rubrics.^24,25^ In addition, candidates can be asked to provide an oral presentation of the case to an examiner who rates their performance on Likert-scale items.^24^ Although candidates’ performance on the consultation, PEF, and oral presentation poorly correlate with each other, it is suggested that this reflects the differing elements of reasoning being assessed by each.^24,27^

Lastly, how OSCE stations are scored may impact candidates’ demonstration of reasoning ability. Checklist-based rubrics often trivialize the complexity of patient encounters and thus may discourage the use of analytical system 2 approaches. Conversely, rating scales that assess component parts of performance (analytic) or overall performance (global) offer improved reliability and validity in capturing a more holistic perspective on candidates’ overall performance.^29^ However, whether analytic or global rating scales differ in their assessment of clinical reasoning remains unclear.^25,27^

Scale issues aside, the challenge for OSCE examiners remains trying to score, through candidate observation, the internal cognitive process of clinical reasoning. Recent work however has provided guidance, suggesting that there are certain observable behaviors demonstrated by candidates which reflect their reasoning processes as shown in Table 3.^30^

**Table 3:** **Observable Behaviors of Clinical Reasoning During Patient Interactions**

Level 1	Student acts	Taking the lead in the conversation
		Recognizing and responding to relevant information
		Specifying symptoms
		Asking specific questions pointing to pathophysiological thinking
		Putting questions in a logical order
		Checking with patients
		Summarizing
		Body language
Level 2	Patient acts	Patient body language, expressions of understanding or confusion
Level 3	Course of the conversation	Students and patients talking at cross purposes, repetition
Level 4	Data gathered and efficiency	Quantity and quality of data gathered
		Speed of data gathering

Based on Haring et al.^31^

## ASSESSING CLINICAL REASONING AT THE “DOES” LEVEL

Since clinical reasoning proficiency in knowledge tests or simulated settings does not automatically transfer to real-life clinical settings, it is critical to continue assessment in the workplace, thereby targeting the top of Miller’s pyramid at the “does” level.^2^ Clinical teachers should assess how learners tackle uncertainty and detect when the reasoning process is derailed by limitations in knowledge or experience, cognitive biases, or inappropriate application of analytic and non-analytic thinking.^5,6^ For example, if novice learners demonstrate non-analytic thinking, clinical teachers should question their reasons for prioritizing certain diagnoses over others. However, advanced learners can apply non-analytic thinking to simpler clinical scenarios. Experts demonstrate higher diagnostic accuracy rates and lower decision making time than novices^30,32^ and skillfully utilize both non-analytic and analytic thinking.^7^ Therefore, learners will benefit when expert clinical teachers think out loud as they develop diagnostic hypotheses.

Clinical teachers routinely observe learners to assess their clinical skills; however, such assessment is often informal, in the moment and impression-based rather than systematic. Moreover, it is often the end point that is assessed rather than the process of reasoning. While summative assessment can determine whether learners have achieved expected competencies, formative assessment fosters a climate of assessment for learning. Frameworks that allow for formative systematic assessment of clinical reasoning are therefore valuable and exemplars are described below.

**Figure 2:**
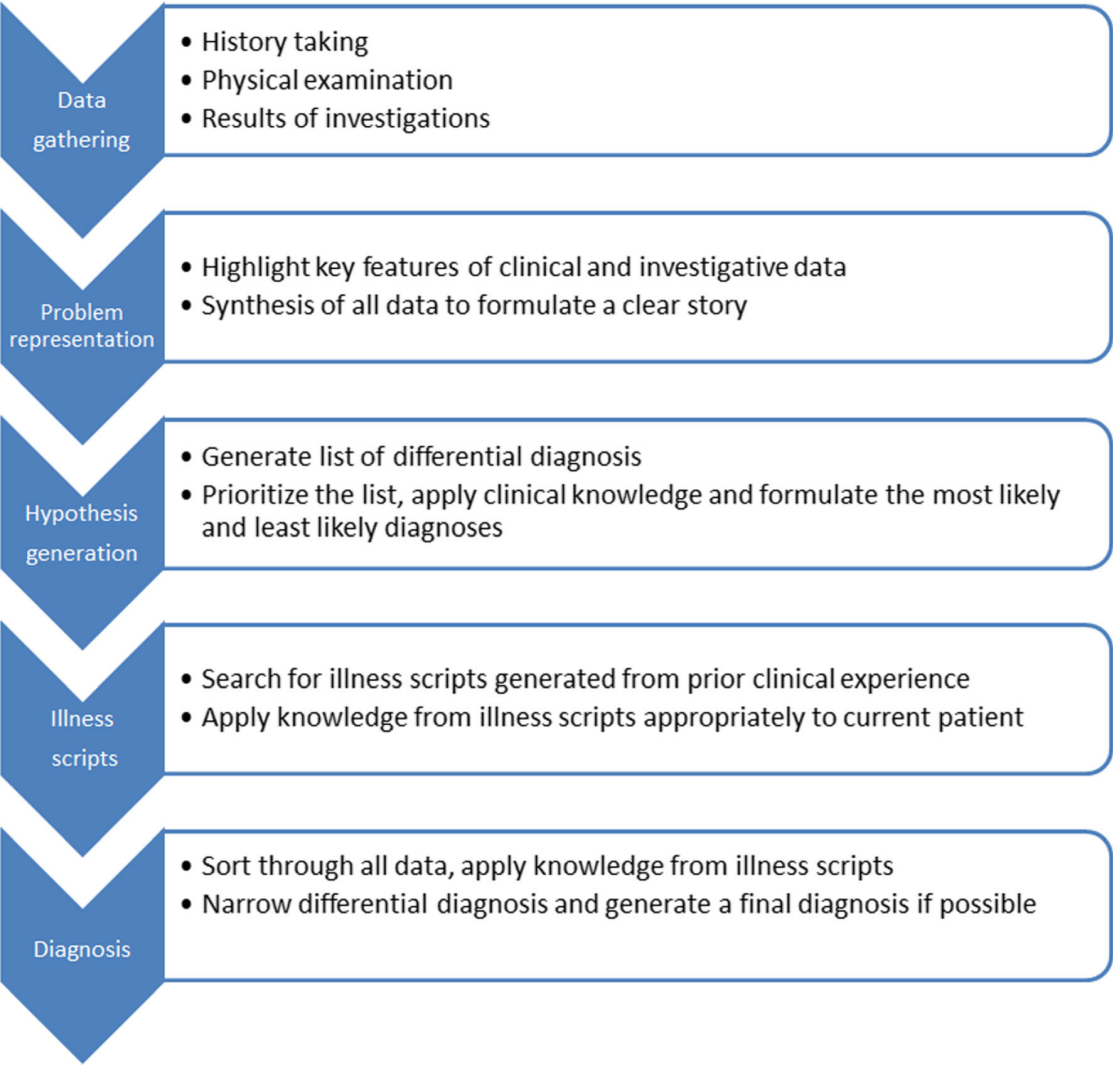
**Steps and strategies for clinical reasoning (supplied also as a .tif file). Adapted from Bowen JL. Educational strategies to promote clinical diagnostic reasoning.*N Engl J Med*. 2006;355(21):2217–25.**

*Bowen’s framework* lists sequential steps that can be demonstrated and assessed by clinical teachers as shown in Figure 2.^33^ These include data gathering (history, examination findings, results of investigations), summarizing key features of the case (problem representation), generating differential diagnoses (diagnostic hypothesis), applying prior knowledge (illness scripts), and final diagnosis.^33^ The *assessment of reasoning tool* similarly describes a five-component assessment process (hypothesis-directed data collection, problem representation, prioritized differential diagnosis, high-value testing, and metacognition) with a simple scoring matrix that rates the learner’s degree of mastery on each component.^34^ The *IDEA framework* moves beyond observed assessments and instead evaluates the clinician’s written documentation for evidence of reasoning across four elements: interpretive summary, differential diagnosis, explanation of reasoning, and alternative diagnoses considered.^35^

Finally, the *one-minute preceptor* model offers a simple and time-efficient framework for formative assessment of clinical reasoning during short case presentations.^36,37^ Learners should develop the skills to synthesize all clinical clues from the history and physical examination, generate an appropriate differential diagnosis, and commit to the most likely diagnosis. Teachers can then pose questions exploring their learners’ skills in diagnostic hypothesis generation, investigative intent, and management planning, seeking their justifications for each. “Getting learners to make a commitment” requires “what” questions and “probing for supportive evidence” requires “how” or “why” questions. “Teaching general rules” assesses how well learners can compare and contrast similar presentations for different patients. Assessment is only meaningful when learners receive ongoing feedback on accuracy of their diagnostic reasoning processes and errors resulting from inappropriate use of non-analytic reasoning and this is achieved through “tell them what they did right” and “correct errors gently”. These steps are depicted in Table 4 in relation to corresponding steps of Bowen’s model with potential methods of assessment for each stage.

**Table 4:** **Clinical reasoning steps based on Bowen's model, potential methods of assessment for each step and corresponding one-minute preceptor strategies**

Clinical reasoning step from Bowen’s model	Potential assessment methods	Corresponding strategies from the one-minute preceptor model
Data acquisition	Direct observation of patient encounter to assess history taking and physical exam skills	
	Case presentation: does the detailed presentation of history and physical exam contain important information?	
Accurate problem representation	Direct observation: questions (pertinent positives and negatives) posed during history taking, targeted physical examination	Getting to a commitment
	Case presentation:	
	-Organization of presentation	
	-Conciseness and accuracy of summary statement	
Generation of hypothesis	Chart-stimulated recall	Probe for supportive evidence
	Case presentation: -Formulation of differential diagnosis linked to clinical data -Prioritization of diagnoses	
	Direct observation	
	-Questions posed to patients	
	-Targeted physical exam	
	Questioning to explore reasons for selection of differential diagnoses	
Selection of illness scripts	Chart-stimulated recall -Explanation of assessment and plans in case write ups	Probe for supportive evidence
	Questioning -Assess application of clinical knowledge -Compare and contrast illness scripts developed by teachers	Teach general rules
	Think out loud -Steps taken to generate and narrow diagnostic hypotheses	
Diagnosis	Case presentation -Specific diagnosis reached	Provide feedback
	Questioning -Narrow differential diagnosis	-Tell learners about appropriate use of analytic and non-analytic reasoning -Gently point out errors

## CONCLUSIONS

This article describes a range of clinical reasoning assessment methods that clinical teachers can use across all four levels of Miller’s pyramid. Although this article has not focused on strategies to help address identified deficiencies in reasoning ability, other authors have developed helpful guidelines in this regard.^38^

As with all areas of assessment, no one level or assessment tool should take precedence and clinical teachers should be prepared and trained to assess from knowledge through to performance using multiple methods to gain a more accurate picture of their learners’ skills. The challenge of case specificity also requires teachers to repeatedly sample and test reasoning ability across different clinical contexts.

Clinical reasoning is a core skill that learners must master to develop accurate diagnostic hypotheses and provide high-quality patient care. It requires a strong knowledge base to allow learners to build illness scripts which can help expedite diagnostic hypothesis generation. As it is a critical step that synthesizes disparate data from history, physical examination, and investigations into a coherent and cogent clinical story, teachers cannot assume that their learners are applying sound reasoning skills when generating differential diagnoses or making management decisions. Enhancing the skills of clinical teachers in assessment across multiple levels of Miller’s pyramid, as well as recognizing and addressing cognitive biases, is therefore key in facilitating excellence in patient care.
